# Immune and inflammatory response in pigs during acute influenza caused by H1N1 swine influenza virus

**DOI:** 10.1007/s00705-014-2116-1

**Published:** 2014-05-21

**Authors:** Małgorzata Pomorska-Mól, Iwona Markowska-Daniel, Krzysztof Kwit, Ewelina Czyżewska, Arkadiusz Dors, Jarosław Rachubik, Zygmunt Pejsak

**Affiliations:** 1 Department of Swine Diseases, National Veterinary Research Institute, Partyzantów 57, 24-100 Pulawy, Poland; 2 Department of Radiobiology, National Veterinary Research Institute, 24-100 Pulawy, Poland

## Abstract

Swine influenza (SI) is an acute respiratory disease of pigs, caused by swine influenza virus (SIV). Little is known about the inflammatory response in the lung during acute SI and its correlation with clinical signs or lung pathology. Moreover, until now there has been a limited amount of data available on the relationship between the concentrations of pro- and anti-inflammatory cytokines in the lungs and the serum concentration of acute-phase proteins (APPs) in SIV-infected pigs. In the present study, the porcine inflammatory and immune responses during acute influenza caused by H1N1 SIV (SwH1N1) were studied. Nine pigs were infected intratracheally, and five served as controls. Antibodies against SIV were measured by haemagglutination inhibition assay, and the influenza-virus-specific T-cell response was measured using a proliferation assay. C-reactive protein (CRP), haptoglobin (Hp), serum amyloid A (SAA), and pig major acute-phase protein (Pig-MAP) the concentrations in serum and concentration of IL-1β, IL-6, IL-8, IL-10, TNF-α and IFN-γ in lung tissues were measured using commercial ELISAs.

Typical clinical signs and an intensive local and systemic inflammatory response were observed after SwH1N1 inoculation. The concentrations of CRP, Hp and SAA increased significantly in pigs with acute SI before a specific immune response was detected. The correlation found between the SAA level in serum and lung scores makes SAA a potentially useful marker for *in vivo* assessment of lung pathology. Because a correlation between the local IL-1β, IL-8 and TNF-α concentration and lung pathology has been observed, we hypothesize that these cytokines are involved in the induction of lung lesions during SI. A positive correlation was also observed between the concentration of IFN-γ in the lungs and clinical signs. No significant relationships between cytokine concentration and APP response were found.

Swine influenza virus (SIV) is the cause of an acute respiratory disease in swine called swine influenza (SI) [1]. SI is characterized by anorexia, fever, dyspnea, coughing and nasal discharge. Infection with SIV generally results in acute inflammation and an immune response that limits viral spread and promotes complete clearance from the lung within 7-10 days [1]. Humoral immunity to infection is mediated by antibodies to the viral surface antigens hemagglutinin (HA) and neuraminidase (NA). In addition to humoral immunity, the T-cell response is also important in defense against SIV and plays a major role in the clearance of virus from the lungs.

To date, little is known about the inflammatory response in particular parts of the lungs during acute SI in pigs. Knowledge about the relationship between local cytokine responses in the respiratory tract of SIV-infected pigs and clinical signs or lung pathology is also limited. Moreover, until now there has been a limited amount of data available on the relationship between the concentrations of pro- and anti-inflammatory cytokines in the lungs and the serum concentration of acute-phase proteins (APP) in SIV-infected pigs.

The acute-phase response is an early response that is mediated by cytokines and involves local and systemic reactions, including changes in the serum concentrations of APP [2]. The clinical utility of APP measurements (e.g., for discriminating between bacterial and viral infections, monitoring of treatment efficacy, or use as prognostic markers) has been extensively studied in human patients [3, 4]. A similar diagnostic value of APP has been proposed in veterinary medicine [2].

In the present study, we examined acute H1N1 SIV infection in pigs with respect to clinical signs, pathology and local and systemic immune responses. The relationship between the intensity of local cytokine secretion and clinical and pathological changes, as well as between concentrations of investigated APPs in serum and changes in the lungs, were analyzed. Furthermore, the association between the local cytokine concentration and the systemic APP response was also investigated.

## Materials and methods

### Animals

Fourteen 6-week-old pigs were obtained from a healthy herd and were shown prior to the start of the study to be both influenza A virus (nasal swabs) and antibody (blood) negative by real-time reverse transcription (RRT)-PCR of the matrix (M) gene and haemagglutination inhibition assay (HI), respectively.

The herd was seronegative for porcine reproductive and respiratory syndrome virus and pseudorabies virus. No evidence of pleuropneumonia, streptococcosis or atrophic rhinitis was recorded. Animals were divided randomly into two groups: a mock (PBS)-infected control group (n = 5) and a group infected by intratracheal inoculation (IT) (n = 9 each).

During the study, pigs were housed at the BSL3 animal facility of the National Veterinary Research Institute (Poland) in two isolated units. Animal use and handling protocols were approved by the local ethics commission (University of Life Sciences, Lublin).

### Preparation of virus inoculum

SIV A/sw/Poland/KPR9/2004 (subtype H1N1) (hereafter referred to as SwH1N1) isolated from the lungs of a pig with acute SI was used for inoculation. The stock represented the third passage in eggs. The virus concentration was determined in Madin-Darby canine kidney (MDCK) cells.

### Experimental design

On day 0, nine pigs were inoculated with SwH1N1. Inoculations of 2 × 10^7.0^ TCID_50_ of virus in 3 ml of phosphate-buffered saline (PBS) per pig were given IT (using a laryngoscope). Five mock-inoculated pigs served as controls. Three infected pigs were euthanized 4, 7 and 10 days post-inoculation (dpi). Pigs from the control group were euthanized at 10 dpi. Necropsy was performed immediately.

Rectal temperatures were measured daily, and clinical signs of disease were recorded. Pigs were observed and scored for respiratory signs as follows: respiratory rate, 0 – normal, 1 – slightly elevated, 2 – moderately elevated, slight abdominal breathing, 3 – clearly elevated, distinct abdominal breathing; nasal discharge, 0 – absent, 1 – present; coughing, 0 – absent, 1 – present; sneezing 0 – absent, 1 – present. All scores per category are accumulated for a total clinical score (CS) of each individual pig (0-6). Fever was defined as a rise in body temperature to 40 °C or above.

Blood samples and nasal swabs were collected at −7, 0, 1, 2, 3, 4, 5, 7 and 10 dpi. Serum was stored at −80 °C for further analysis. Necropsy was done on each animal, with special emphasis on the respiratory tract. Gross lung lesions were examined for the presence of multifocal consolidation, and when present, extension was recorded. Samples from lung (all lobes separately) and tracheas were collected for viral RNA extraction and estimation of cytokine concentrations.

Lung lesions were scored using the method developed by Madec and Kobisch [5] according to the following scheme: 0 points, no lesion; 1 point, lesions affecting <25 % of the lobe surface; 2 points, lesions affecting 25-49 % of the lobe surface; 3 points, lesions affecting 50-74 % of the lobe surface; and 4 points, lesions affecting >75 % of the lobe surface. All recorded scores were then added together to determine the final visual lung score for each pig, ranging from 0 to 28.

### Laboratory examination

#### Swabs and tissue samples

An M gene RRT-PCR was used for detection of SIV as described previously [6]. RRT-PCR was performed using a one-step RT-PCR kit (QIAGEN, Valencia, CA). The oligonucleotide sequences were specific for the M gene region of known European SI A viruses, as well as pdm-like H1N1 influenza A virus. Samples with a Ct value <30 were considered positive, samples with a Ct value of 30-35 and with sigmoidal/logarithmic appearance were considered weakly positive, and samples with a Ct value >35 were considered negative.

#### Haemaglutinin inhibition assay (HI)

Antibodies against SIV were measured using an HI assay, performed according to the standard procedure using 0.5 % chicken erythrocytes and 4 HA units of SwH1N1, H3N2 (A/Sw/Flanders/1/98) or H1N2 (A/Sw/Granstedt/2004) virus. All sera were tested in serial twofold dilutions, starting at 1:20.

#### Lymphocyte proliferation assay

The lymphocyte proliferation assay to measure influenza-specific T-cell responses of pigs was performed at 0, 4, 7 and 10 dpi as described previously [7]. Briefly, PBMCs were isolated from blood samples by centrifugation onto Histopaque 1.077 (Sigma, USA) and were washed twice with PBS. The isolated PBMCs were seeded in plastic vials at a density of 1 × 10^6^ viable cells per vial in 1 ml of medium (RPMI 1640 containing 10 % fetal bovine serum, 2 mM L-glutamine and 1 % of antibiotic-antimycotic solution). For analysis of cellular responses, PBMCs were restimulated *in vitro* with 50 µl of medium containing live SwH1N1 virus (10^7.0^ TCID_50_/50 µl). Control cells were incubated without the virus (mock-control) or with 5 µg/ml of concanavalin A (Con-A) (vitality control). All samples were analysed in triplicate.

After 72 hours of incubation at 37 °C in a 5 % CO_2_ atmosphere, the cultures were pulsed with 0.5 µCi [^3^H]-thymidine (MP Biomedicals, USA). After 18 hours, cells were harvested, and the incorporated radioactivity was measured in a liquid scintillation counter (Tri-Carb 2500TR, Packard, USA). Proliferation was expressed as a stimulation index (SIx), calculated as the number of counts per minute (cpm) for SwH1N1-stimulated cells divided by the number of cpm for mock-stimulated cells (in each case, taking the mean of triplicate readings). Based on the SIx values of the control group (mean plus 3 times the standard deviation), a SIx value equal to or higher than 1.55 was considered positive.

#### APP determination in serum samples

For determination of APP, commercial ELISAs were used according to the manufacturer’s recommendations (Pig C-Reactive Protein ELISA and Pig Haptoglobin ELISA from Life Diagnostics, Inc., USA; PigMAP KIT ELISA from PigCHAMP Pro Europa S.A, Spain; Phase Serum Amyloid A Assay from Tridelta Development Ltd., County Kildare, Ireland). Prior to analysis, serum samples were diluted as follows: 1:2000 for CRP, 1:35,000 for Hp, 1:500 for SAA and 1:1000 for PigMAP.

##### Cytokine concentration in the lungs

Lung tissue were collected from control and infected pigs during necropsy and prepared in PBS (pH 7.4) [8]. One gram of the trachea or lungs tissue (all lobes separately) was suspended in 1 ml of PBS (1:1 w/v) and frozen before being homogenized. After homogenization, the samples were centrifuged at 12,000 rpm for 10 min. The supernatants were collected and stored at −80 °C before cytokine analysis was performed using porcine cytokine ELISA kits. The ELISA kits specific for porcine IL-8, IL-10, IFN-γ and TNF-α were purchased from Invitrogen Corporation (Camarillo, USA), and those used for porcine IL-1β were purchased from RayBiotech, Inc. (Norcross, USA). The concentration of IL-6 was determined using an IL-6 Pig ELISA Kit from Abcam (Cambridge, UK). The detection limits of kits are as follows: 6 pg/ml (IL-1β), 45 pg/ml (IL-6), 10 pg/ml (IL-8), 3 pg/ml (IL-10), 2 pg/ml (IFN-γ) and 3 pg/ml (TNF-α). All assays were performed according to the manufacturers’ protocols. All of the samples were tested in duplicate. The quantity of the cytokines was calculated based on a standard curve for each cytokine using FindGraph software. For statistical analysis, levels lower than the detection limits were set to detection limit (in pg/ml) minus 1 (1 pg/ml for IFN-γ, 9 pg/ml for IL-8, and 2 pg/ml for IL-10).

### Statistical analysis

The data were subjected to the Shapiro-Wilk test of normality and Levene’s test of equal variances with STATISTICA 8.0 (StatSoft). Differences between means were tested for statistical significance by a nonparametric Kruskal-Wallis test. The Friedman test was used to compare observations repeated on the same subjects. Comparisons between infected and control groups at each time point were assessed using the Mann-Whitney U test. For analysis of correlation, the Spearman rank correlation test was used. For all analyses, p < 0.05 was considered significant.

## Results

### Clinical signs and pathological examination

**Fig. 1:**
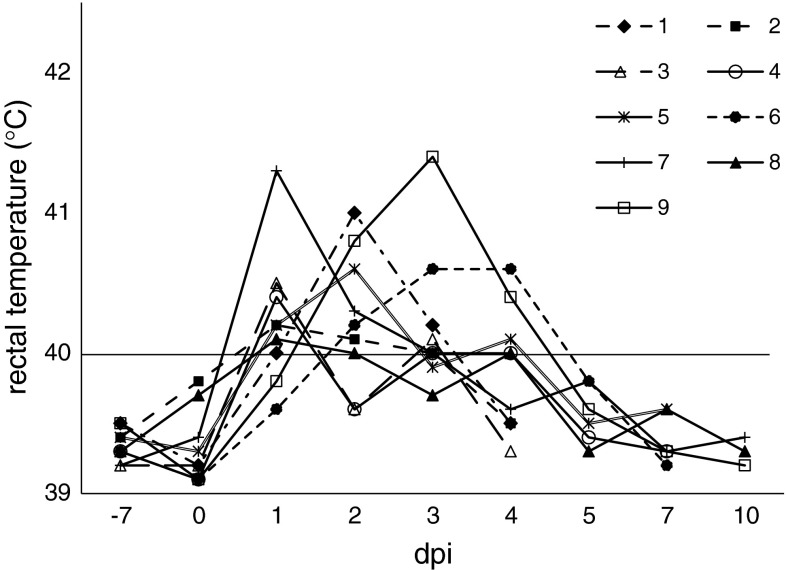
Individual rectal temperatures of pigs inoculated intratracheally with swine influenza virus (H1N1)

All inoculated pigs displayed clinical signs of SI (coughing, sneezing, nasal discharge) and abnormal rectal temperatures between 1 at 4 dpi (Fig. 1). The mean clinical score was 2.66 ± 1.32 (ranging from 1 to 5). In control pigs, no clinical signs of any disease were seen, and rectal temperatures were ≤40 °C.

**Fig. 2:**
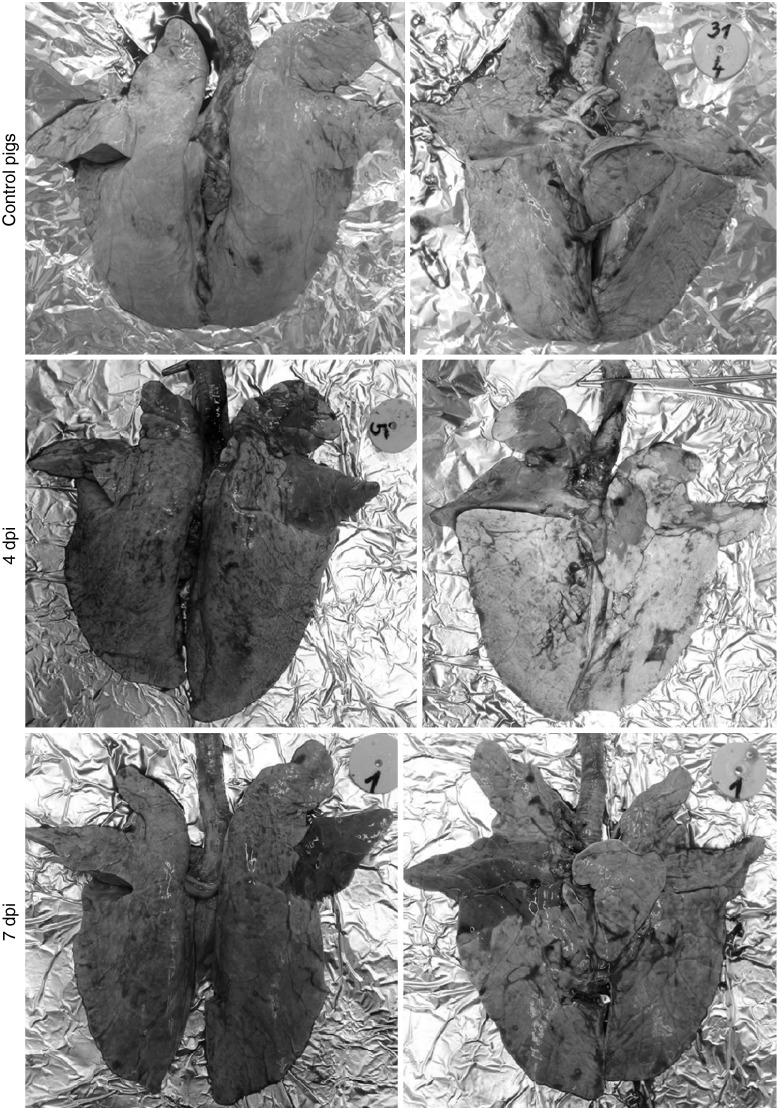
Changes observed at 4 and 7 days post-inoculation (dpi) in the lungs of pigs infected intratracheally with swine influenza virus (H1N1) and in control pigs

Necropsy revealed typical lesions in the lungs of nine out of nine infected pigs. Lung lesions were of variable extent and were predominantly located in the right and left cranial and middle lung lobes. The mean lung score was 5.33 ± 2.06 (range 3-10). There were significant differences in the lung score between pigs necropsied at 4 or 7 dpi and pigs necropsied at 10 dpi (p < 0.05). The typical lesions are shown in Fig. 2. No gross lung lesions were seen in control pigs.

### Presence of SIV in swabs and tissues

RRT-PCR assay revealed positive results in nasal swabs taken between 1 and 7 dpi from all infected pigs (Table 1). In the nasal swabs taken from control pigs, no SIV RNA was found. No viral RNA was detected in swabs at 10 dpi. In infected pigs necropsied at 4 and 7 dpi, the presence of SIV was confirmed for trachea and middle lobes. In four pigs, the occurrence of SIV RNA was also confirmed for apical and accessory lobes. No viral RNA was found in left and right caudal lobes in samples taken at 10 dpi and in control pigs.

**Table 1:** Real-time RT-PCR results for clinical samples (nasal swabs) from pigs inoculated with H1N1 swine influenza virus and control pigs

Animal number	Time postinfection (days)							
	0	1	2	3	4	5	7	10
1	−	−	−	++	++	na	na	na
2	−	+	++	++	++	na	na	na
3	−	+	++	++	++	na	na	na
4	−	−	−	++	++	++	++	na
5	−	−	+	++	++	++	++	na
6	−	+	++	++	++	++	+	na
7	−	+	++	++	+	+	+	−
8	−	+	++	++	++	++	++	−
9	−	−	−	++	+	++	++	−
Controls	−	−	−	−	−	−	−	−

Real-time RT-PCR results are given as ++ (Ct value <30; positive), + (Ct value 30-35; weak positive), − (Ct value >35, negative), na (not applicable)

### Antibody and cellular response against SwH1N1

Five out of six inoculated pigs exhibited SwH1N1-specific antibodies in their serum between 7 and 10 dpi (Table 2). The uninfected pigs had no detectable anti-HA antibodies.

**Table 2:** Individual hemagglutination inhibition titres in serum samples from pigs inoculated with H1N1 swine influenza virus and from control pigs

Animal number	dpi			
	0	4	7	10
1	-	-	na	na
2	-	-	na	na
3	-	-	na	na
4	-	-	20	na
5	-	-	20	na
6	-	-	-	na
7	-	-	20	80
8	-	-	40	160
9	-	-	-	40
Controls	-	-	-	-

A titre below 20 was assigned as “-”

na, not applicable

**Fig. 3:**
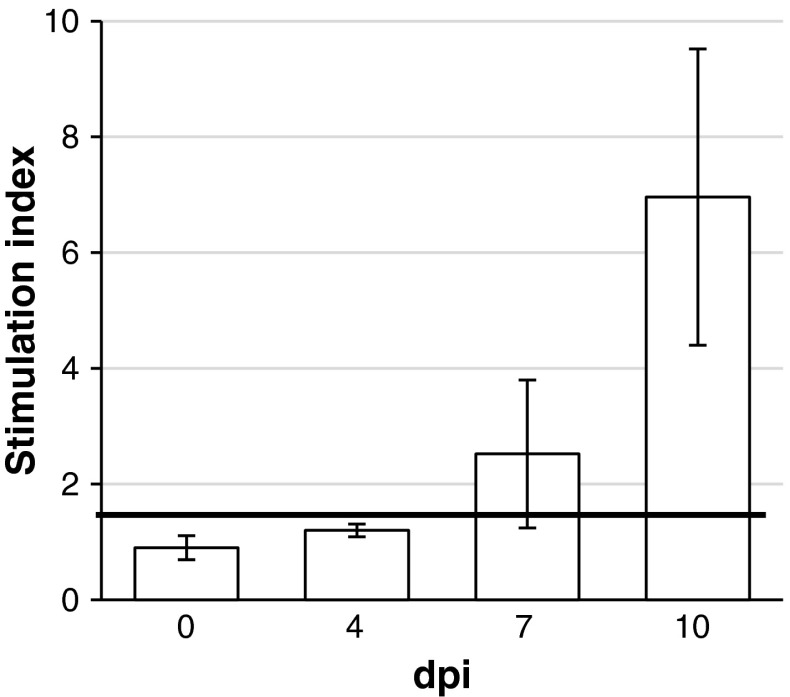
Mean (±SD) stimulation index values in pigs inoculated intratracheally with H1N1 swine influenza virus and in control pigs. The bold line indicates the value considered to represent antigen-specific proliferation

The mean SIx values in control pigs and pigs from experimental groups before inoculation ranged from 0.82 to 1.02. Antigen-specific proliferation was noted for the first time at 7 dpi in four out of six inoculated pigs. At 10 dpi, a specific T cell response was observed in three out of three inoculated pigs. The mean SIx values (±SD) in SwH1N1-inoculated pigs are presented in Fig. 3.

### Acute-phase proteins

**Fig. 4:**
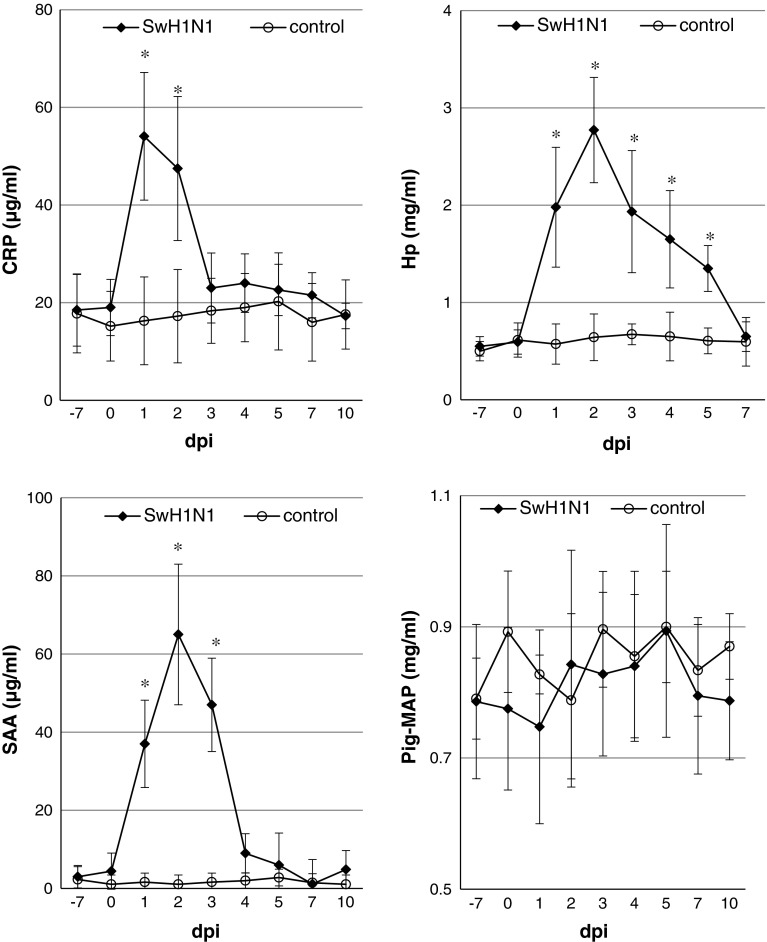
Concentrations of CRP, Hp, SAA and Pig-MAP in serum of pigs before and at various time points after intratracheal infection with H1N1swine influenza virus (mean ± SD). *p < 0.05, significant increase compared to control animals

The concentrations of CRP, Hp and SAA increased significantly after inoculation with SwH1N1, with mean maximum levels from 1 to 2 dpi. The time course of mean APP concentrations (±SD) during the study period is shown in Fig. 4.

The mean concentration of CRP was significantly higher from 1 to 2 dpi than in controls (p < 0.05). The mean maximum concentration reached over 54 μg/ml and was almost 3-fold higher than it was before inoculation. Starting at 3 dpi, the CRP concentration decreased and did not differ significantly between control and infected pigs.

Individual pre-inoculation levels of Hp were found to be below 0.65 mg/ml. The highest individual induced level reached 3.37 mg/ml (at 2 dpi). Changes in serum Hp concentrations were observed in all SwH1N1-inoculated pigs. The mean peak level was over 5-fold higher than at 0 dpi. The levels of Hp remained significantly elevated until 7 dpi (p < 0.05).

The mean concentration of SAA increased significantly during the first 24 h after inoculation. A significant increase in the mean SAA concentration, as compared to the control pigs, was observed from 1 to 3 dpi (p < 0.05). The highest individual concentration of SAA was seen at 2 dpi. The mean peak level reached over 65 μg/ml and was over 20-fold higher than the level on day 0. Starting at 4 dpi, the SAA concentration decreased and did not differ significantly between control and infected pigs. A significant correlation was found between the maximum concentration of SAA in serum and changes in the lungs (R-Spearman = 0.70, p < 0.05).

The concentrations of PigMAP did not differ significantly from those observed in uninfected pigs (p > 0.05).

### Local cytokine response

**Fig. 5:**
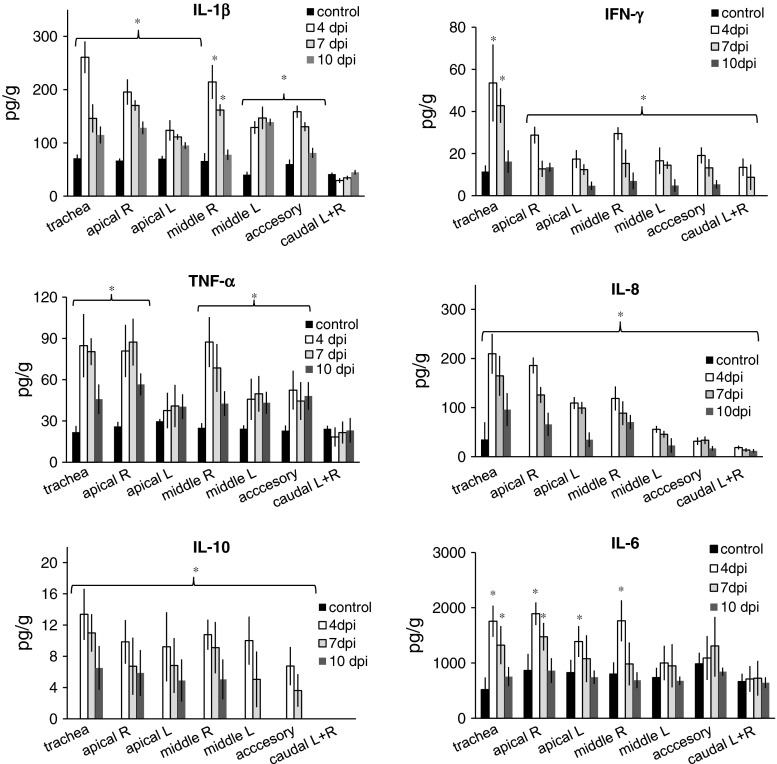
Quantification of cytokines in lungs of control pigs and pigs with acute influenza caused by H1N1 swine influenza virus (mean ± SD). R, right; L, left; *, significant difference compared to the corresponding tissue sample from control pigs

In general, the local concentrations of IL-1β, IL-6, IL-8, IL-10, TNF-α and IFN-γ were increased in SwH1N1-inoculated pigs (Fig. 5). In control pigs, the concentrations of IFN-γ, IL-8 and IL-10 were below the detection limit.

At 4 dpi, concentrations of IL-1β, IL-8, IL-10 and IFN-γ were significantly higher in the lungs of infected pigs than in controls. However, in caudal lobes, the concentration of IL-10 was below the detection limit. The concentrations of IL-6 and TNF-α were significantly higher only in tracheas and cranial parts of the lungs (p < 0.05). No significant differences were found between control and infected pigs with regard to TNF-α level in caudal and left apical lobes and to the IL-6 level in left middle and accessory lobes and both caudal lobes. The highest concentrations of cytokines were generally observed in right apical and middle lobes, which correlated well with the lesions observed during necropsy.

At 7 dpi, the concentrations of most investigated cytokines were also significantly higher in infected pigs than in controls. With regard to IL-6, a significantly higher concentration was observed only in the tracheas and apical lung lobes. In general, the concentrations of cytokines were the highest in the cranial parts of the lungs, while in caudal lobes, they were the lowest.

At 10 dpi, the lung concentrations of cytokines generally decreased compared to 4 dpi, but in some cases, they were still significantly higher than in controls (p < 0.05). Significantly greater concentrations were observed for IL-8 with regard to all lobes; for IL-1β, IL-10, IFN-γ and TNF-α, with regard to cranial parts of the lungs (p < 0.05). No significant differences between local concentrations of IL-6 in infected and control animals were found for any of the lung lobes (p > 0.05).

In summary, the highest concentrations of cytokines were observed on 4 dpi in the tracheas and cranial parts of the lungs (apical, middle and accessory lobes). These results correlated well with localization of gross lung lesions observed in infected pigs. Significant correlations were found between lung concentrations of IL-1β, IL-8 and TNF-α and pathological changes in the lungs (R-Spearman = 0.72; 0.68 and 0.77, respectively; p < 0.05). A positive correlation was also found between concentrations of IFN-γ in the lungs and clinical signs (R-Spearman = 0.69, p < 0.05). No significant correlation between local cytokine concentration and systemic APP response was found (p > 0.05).

## Discussion

In present study, we examined pigs with acute H1N1 SIV with respect to clinical signs, pathology and local and systemic immune response during the first 10 dpi. In addition, the correlation between serum APP concentrations and lung scores, as well as between cytokine concentrations in the parenchyma of the lungs and clinical and lung scores, were analyzed. The relationship between local cytokine concentrations and systemic acute-phase protein (APP) response was also investigated.

Pigs can serve as an animal model in studies of influenza pathogenesis. The clinical manifestations and course of the influenza in pigs is similar to those observed in humans [9]. Moreover, the porcine lung has significant similarities to human lungs (structure of the tracheobronchial tree, physiology, patterns of glycoprotein synthesis) [9]. In addition, the cytokine responses in bronchoalveolar lavage fluid in SIV-infected pigs are identical to those in humans [10].

In the present study, typical acute SI was observed in inoculated pigs, and lung lesions were found in all SwH1N1-infected pigs. The lung lesions observed at 4 and 7 dpi were more severe than those observed at 10 dpi (p < 0.05). Specific anti-HA antibodies were detected at 7 and 10 dpi in inoculated pigs. Serum anti-HA antibodies are the ones most commonly measured to determine the level of protection against influenza virus. Usually, they can be detected at 7-10 dpi [11]. It has been shown previously that cell-mediated immunity plays a role in recovery from influenza virus infection and may also prevent influenza-associated complications, but it does not seem to contribute significantly in preventing infection [1]. T-cell immune responses are also critical for the complete clearance of the virus [1]. Our results showed that SIV-specific proliferation appeared quite early. Starting from 7 dpi, an antigen-specific response was seen in four out of six SIV-infected pigs. The kinetics of the T-cell proliferative response agrees in general with the result of Larsen et al. [12], who found that an antigen-specific T-cell response in pigs with acute influenza could be detected starting at 7 dpi, reaching peak levels at 14 dpi.

After intratracheal inoculation of pigs with SwH1N1, variations in the kinetics of the APP response were observed. CRP and SAA were significantly induced at the very early stage of infection (from 1 to 2 and 3 dpi, respectively). The Hp response was more protracted compared to that of CRP and SAA, with a significant increase from 1 to 5 dpi (p < 0.05). The concentration of pigMAP remained unchanged to the end of the study (p > 0.05). No significant correlation between the local cytokine concentration and the APP response was found (p > 0.05).

Earlier studies in pigs have described the kinetics of the APP response in the course of various diseases, including acute and subclinical SI [13, 14]. Barbé et al. [13] investigated the CRP and Hp response associated with acute SI during first the 5 dpi. In general, the serum concentrations of CRP and Hp peaked at 2 dpi, but in contrast to our study, no significant differences were found between infected and control pigs. The difference in the APP profiles may be due to the different virus used in the study of Barbé et al. [13] or the smaller number of pigs per group. An increase of CRP and Hp in pigs was also reported by Brookes et al. [15] during clinical influenza caused by pandemic (H1N1) 2009 virus. The CRP levels peaked at 4 dpi, while Hp responses were more protracted, peaking at 9-11 dpi [15]. In our experiment, the maximum CRP concentration was observed earlier, at 1 dpi. This could be a consequence of using a different route of inoculation (intranasal in Ref. 15).

SAA has proven potentially useful as a bacterial infection marker in pigs, but knowledge about the SAA response in viral diseases, including SI, is limited. Similar to what has been observed in previous studies in humans, horses and pigs [14, 16, 17], a significant increase in serum SAA was found in the present study. Moreover, a correlation between the SAA serum concentration and lung pathology was observed.

In the present study, in accordance with our previous reports [7, 14], no significant response of PigMAP was seen. Also, no significant increase in the concentration of PigMAP was observed with other viral diseases in pigs (porcine reproductive and respiratory syndrome, pseudorabies) [18].

Cytokines also play an important role in the immunopathology of SIV. Their concentrations in bronchoalveolar lavage fluid have been correlated with viral replication and clinical signs as well as increased lung lesions and neutrophil infiltration [19, 20]. In our study, the concentrations of all cytokines were elevated in the tracheas and lungs of the infected pigs compared to the controls. The highest concentrations of the investigated cytokines were observed on 4 dpi in the tracheas and cranial parts of the lungs. A significant correlation was found between lung lesions and lung concentrations of IL-1β, IL-8 and TNF-α. Previous reports [19, 21] also have shown a positive correlation between an elevated level of IL-1β and neutrophil recruitment to the lungs, which may lead to the more-severe inflammation. The higher concentration of IL-8 may also have contributed to the more-severe lesions. In agreement with Barbé et al. [13], a significant correlation between the concentration of IFN-γ and clinical signs was observed in our experiment. In the lungs of control pigs, the level of IFN-γ was below the detection limit. Gamma interferon is produced during the early stages of an infection, and its major functions are activation of macrophages, differentiation of Th1 from T cells and control of intracellular pathogens [22]. Similarly to the results obtained by Jo et al. [8], we found significantly higher concentration of TNF-α in the tracheas and cranial parts of the lungs of infected pigs at 4 and 7 dpi. According to Damjanovic et al. [23], TNF-α is critically required for negatively regulating the extent of lung immunopathology during acute influenza virus infection. The significantly higher concentrations of IL-6 in lung tissues were observed mainly in the early stage of infection (mostly at 4 dpi). Lauder et al. [24] reported that IL-6 has an essential role in orchestrating anti-influenza immunity through its ability to limit inflammation, promote protective adaptive immunity, and prevent fatal immunopathology. The early secretion of this cytokine may therefore constitute an important line of defense against a fatal course of influenza.

The cytokines associated with acute SI in pigs were also studied previously by Van Reeth et al. [20]. Induction of IL-1 and TNF-α, but not IL-8, was observed. In that study, however, the cytokines were measured in bronchoalveolar lavage fluid rather than lung tissue. The difference in cytokine profiles may be also due to the different viruses used for inoculation.

The role of IL-10 during acute influenza appears to be contradictory. Sun et al. [25] found that inhibition of IL-10 signaling during ongoing influenza resulted in increased inflammation and decreased survival, whereas McKinstry et al. [26] reported that inhibition of IL-10 signaling before infection enhanced viral clearance and increased survival. In the present study, the highest levels of IL-10 were detected in the tracheas and cranial parts of the lungs; however, no significant correlation was found between lung pathology and the local cytokine level.

## Conclusions

In the present study, the typical clinical signs of acute SI were observed in IT-inoculated pigs. Furthermore, an intensive local and systemic inflammatory response was induced by infection. Surprisingly, no evident relationship between lung pathology and clinical signs was found. The serum concentrations of CRP, Hp and SAA increased significantly in pigs with acute SI before a specific immune response was detected. The significant correlation found between SAA concentration in serum and lung scores makes this APP a potentially useful marker in the *in vivo* assessment of lung pathology. SwH1N1 virus induces local pro-inflammatory and immune-suppressive cytokines responses during acute influenza. Because a positive correlation between local cytokine concentration and lung pathology has been observed, we hypothesize that local production of IL-1β, IL-8 and TNF-α is involved in the induction of lung lesions during influenza infection.
